# Differential Cortical and Subcortical Activations during Different Stages of Muscle Control: A Functional Magnetic Resonance Imaging Study

**DOI:** 10.3390/brainsci14040404

**Published:** 2024-04-20

**Authors:** Yu Peng, Zhaoxin Wang

**Affiliations:** 1 Key Laboratory of Brain Functional Genomics (MOE & STCSM), Institute of Cognitive Neuroscience, School of Psychology and Cognitive Science, East China Normal University, Shanghai 200062, China; pengyu159t@163.com; 2 Shanghai Changning Mental Health Center, Shanghai 200355, China; 3 Shanghai Key Laboratory of Magnetic Resonance, East China Normal University, Shanghai 200062, China

**Keywords:** basal ganglia, caudate nucleus, putamen, subthalamus nucleus, muscle contraction, muscle relaxation

## Abstract

Movement and muscle control are crucial for the survival of all free-living organisms. This study aimed to explore differential patterns of cortical and subcortical activation across different stages of muscle control using functional magnetic resonance imaging (fMRI). An event-related design was employed. In each trial, participants (*n* = 10) were instructed to gently press a button with their right index finger, hold it naturally for several seconds, and then relax the finger. Neural activation in these temporally separated stages was analyzed using a General Linear Model. Our findings revealed that a widely distributed cortical network, including the supplementary motor area and insula, was implicated not only in the pressing stage, but also in the relaxation stage, while only parts of the network were involved in the steady holding stage. Moreover, supporting the direct/indirect pathway model of the subcortical basal ganglia, their substructures played distinct roles in different stages of muscle control. The caudate nucleus exhibited greater involvement in muscle contraction, whereas the putamen demonstrated a stronger association with muscle relaxation; both structures were implicated in the pressing stage. Furthermore, the subthalamic nucleus was exclusively engaged during the muscle relaxation stage. We conclude that even the control of simple muscle movements involves intricate automatic higher sensory–motor integration at a neural level, particularly when coordinating relative muscle movements, including both muscle contraction and muscle relaxation; the cortical and subcortical regions assume distinct yet coordinated roles across different stages of muscle control.

## 1. Introduction

Movement is essential for the survival of all free-living organisms [1]. All forms of movement are a result of changes in the state of muscles, including muscle contraction, holding, and relaxation [2]. The control of muscles by the brain in these different stages poses an intriguing question.

However, previous imaging studies have predominantly focused on the neural correlates of force generation (i.e., muscle contraction) [3,4,5,6,7,8] or the fine modulation of movement [9,10,11,12]. For recent reviews, please refer to Farina and Gandevia [13] and Hardwick et al. [14]. The involvement of the key cortical areas, such as the pre-supplementary motor area (pre-SMA), SMA, premotor area (PMA), and primary motor cortex (M1), in muscle contraction is widely acknowledged. Nevertheless, inconsistencies in the subcortical areas still exist. For example, a recent meta-analysis revealed that cortical activation spans the sensorimotor area and PMA, etc., while subcortical clusters were identified in the bilateral thalamus, putamen, and cerebellum [14]. Conversely, Spraker et al. reported that there was robust bilateral activation in the caudate for precisely controlled force generation compared with rest [15].

The neural correlates of muscle relaxation have been relatively under-studied. Interestingly, Toma et al. initially reported that muscles do not relax through the simple cessation of projection neuron activity in M1 [16]. In fact, relaxation is an active process requiring a degree of cortical activation similar to or even greater and more widespread than that of muscle contraction [2,8,17]. For a recent review, refer to Kato et al. [2]. Abnormalities in cortical activity during muscle relaxation have been observed in aging [18], patients with Parkinson’s disease [19], and writer’s cramp [20]. Therefore, an understanding of the mechanisms of muscle relaxation is just as important as comprehending those involved with muscle contraction [2]. However, although the involvement of the M1/SMA/PMA has been revealed in muscle relaxation, the subcortical correlates remain under-studied.

Moreover, the neural correlates of muscle holding have also been under-studied. To the best of our knowledge, only three studies have investigated the neural correlates of muscle holding [3,9,21]. Among them, Ehrsson et al. have studied force adjustments to external perturbations [3]. In Kuhtz-Buschbeck et al.’s study [9], fMRI data were collected from eight healthy male volunteers with a 1.5-Tesla MR system, where subjects alternated between rest periods and precision grip-hold periods (30 s). Compared to the resting period, only the left primary sensorimotor cortex (M1/S1), the left intraparietal regions, and a small right posterior parietal area were active [9]. In Vaillancourt et al.’s study, the hold task required subjects to generate steady-state force for a 30 s block [21]. However, the results of the steady-state force condition were taken as a control, and were not illustrated in detail, although activation in specific brain regions, such as the caudate, SMA/anterior cingulate cortex (ACC), etc., were reported in their figures, with a voxel-wise threshold of *p* < 0.005 and k = 5. Critically, these studies analyzed the mean signal during the entire process, encompassing both muscle contraction, holding, and relaxation. That is to say that the potential differential neural correlates of the three stages were not elucidated. However, a common finding was that brain activation related to holding was very weak, which may explain why it has not been thoroughly investigated.

Therefore, although tremendous work has been conducted on the neural correlates of muscle control, and research has proven the crucial roles of the M1/PMA/SMA, there remains a gap in the literature regarding the potential differential cortical activation among different stages of muscle control, i.e., muscle contraction, holding, and relaxation. Furthermore, conclusive findings concerning the subcortical regions during these stages are lacking. Therefore, further studies are warranted to address these gaps.

The aims of the present study were to examine potential differences in cortical and subcortical activation patterns during different stages of muscle control, i.e., muscle contraction, steady holding, and relaxation. To achieve this objective, participants were instructed to press a key and hold it for a while, and then relax, thus allowing for the temporal separation of these stages of muscle control and enabling examination of the underlying neural correlates. As this is a very simple task and no complicated manipulation is needed, the cognitive load was kept at a minimal level to isolate the neural correlates of muscle control as much as possible. Based on previous findings, we hypothesized that both the cortical and subcortical regions exhibit differential activity across these different distinct stages. The subcortical basal ganglia are of particular interest. It has been proposed that the basal ganglia are involved in motor modulation [22], and two major pathways have been identified [22,23,24,25,26,27], i.e., the direct pathway and the indirect pathway. There is also a third hyperdirect pathway involving neurons that travel directly from the cerebral cortex to the STN, bypassing the striatum. This pathway has a shorter conduction time than effects conveyed through the striatum, and therefore provides rapid inhibition for action suppression [24] and non-motor suppression [28]. It is expected that this pathway would be less engaged in the natural relaxation of muscles in this study. The direct pathway facilitates the intended movements, while the indirect pathway inhibits the neural responses of the thalamus, making excitation of the motor cortex less likely, hence inhibiting action. Studies of Parkinson’s disease have shown that patients are characterized by slowness of movement (bradykinesia), possibly due to dysfunction in the direct pathway [22,23,24,25,26,27,29,30]. Conversely, Huntington’s disease is associated with rapid, jerky motions with no clear purpose, possibly due to dysfunction in the indirect pathway [22]. Moreover, the specific roles of the two subregions of the striatum (i.e., caudate and putamen) in voluntary motor control are not fully understood, but may exhibit differences. For example, activation in the putamen, as opposed to the caudate, has been associated with successful inhibition [31,32,33,34], including inhibiting counting [28]. Note that in patients with Parkinson’s disease, the loss of dopamine occurs predominantly in the posterior putamen [35]. Conversely, activation in the caudate, rather than the putamen, has been linked to actions such as holding tasks [21] and response executions [36,37]. Therefore, we hypothesized that there are distinct patterns of cortical and subcortical activation between muscle contraction, holding, and relaxation, especially in the basal ganglia.

## 2. Materials and Methods

### 2.1. Participants

Ten native Chinese students (3 assigned male at birth, age = 24.5, SD = 1.6) from East China Normal University were recruited. A power analysis was conducted using G*Power version 3.1.9.7 [38] to determine the minimum sample size required to test the study hypothesis. The results indicated that the required sample size to achieve 80% power for detecting a large effect size (1, as this study focused on the contrast of mean signal change vs. 0) at a significance criterion of α = 0.05 was *n* = 10 for a two-tailed *t*-test. The participants were recruited via advertisements and flyers at a local university, with the following inclusion criteria: adults with normal motor functions, right-handedness, normal or corrected-to-normal vision, and normal color perception. The exclusion criteria included a self-reported history of psychiatric or neurological disease, head injury, or drug abuse. After completion of all tasks, participants were debriefed and paid as compensation for their time. This study was carried out in accordance with The Code of Ethics of the World Medical Association. Written informed consent was obtained and the protocol was approved by the University Committee on Human Research Protection of the East China Normal University.

### 2.2. Procedures

An event-related design was implemented. In each trial, a press–hold–relax procedure was employed, with participants instructed to gently press a button on a hand-shaped response box with their right index fingers upon the appearance of a green circle on the screen, to subsequently hold it for 7 s, and to relax their finger when the circle disappeared. The circle appeared for 7 s. The mean accuracy was 94% (SD = 11%), and mean reaction time (RT) was 289 ± 59 ms. (a) This time interval was chosen for two reasons: (i) to minimize habituation while allowing for detection of the blood oxygenation level-dependent (BOLD) signal, and (ii) to prevent participant fatigue. A 17 s blank inter-stimuli-interval (ISI) was followed. There were 4 functional runs, each incorporating 15 trials. (b) Participants were instructed to press the button with their fingers gently, which is based on a previous study in which the SMA/ACC exhibited significantly higher activity during a gentle force condition compared to other conditions, despite weaker contractions of the hand muscles [9]. Stimuli were presented using a goggle system, and responses were collected using a hand-shaped response box (Invivo Co., Gainesville, FL, USA).

### 2.3. Data Collection and Analysis

The scanning was performed using a 3-Tesla Siemens Trio MR scanner, including 4 functional runs and 1 anatomical run. For functional images, 35 axial slices (FOV = 240 × 240 mm^2^, matrix = 64 × 64, in-plane resolution = 3.75 × 3.75 mm^2^, thickness = 4 mm, without gap) covering the whole brain were obtained using a T2*-weighted echo planar imaging (EPI) sequence (TR = 2000 ms, TE = 30 ms, flip angle = 90°). A high-resolution structural image for each participant was also acquired using 3D MRI sequences for anatomical co-registration and normalization (TR = 1900 ms, TE = 3.43 ms, flip angle = 7°, matrix = 256 × 256, FOV = 240 × 240 mm^2^, slice thickness = 1 mm).

SPM12 was adopted for data analysis (Wellcome Department of Cognitive Neurology, London, UK; http://www.fil.ion.ucl.ac.uk/spm/, accessed on 18 April 2024). EPI images were first realigned to the first volume of the first run to correct for head motions. Then, the anatomical image was co-registered with the mean EPI image and segmented, and then normalized parameters were generated and projected to the MNI space. Using these parameters, all EPI data were projected to the MNI space with a 2 × 2 × 2 mm^3^ resolution, and then smoothed using an 8 mm FWHM (full width half maximum) isotropic Gaussian kernel. High-pass temporal filtering with a cut-off of 128 s was also carried out to remove low-frequency drifts [39].

For the first-level analysis, a General Linear Model with three stages (pressing, holding, and relaxation) convolved with the canonical hemodynamic response function (HRF) was applied. Six estimated head movement parameters were included in the design matrix to reduce the residual effects of head motion. Parameter estimates were then put into the second-level group random-effects analysis with a one-sample *t*-test. The voxel-wise threshold was set at *p* < 0.001 and k > 80 for the pressing and relaxation stages, while it was set at *p* < 0.005 and k > 80 for the holding stage to balance Types I and II errors as its activation was very weak [40]. The template of each brain region was adapted from Automated Anatomical Labeling (AAL), and small volume corrections (SVC) for multiple comparisons were performed using 10 mm spheres centered at each activation map. The mean time course of the peak voxel in regions of interest for each condition was drawn using AFNI (Analysis of Functional NeuroImages, http://afni.nimh.nih.gov/).

## 3. Results

**Figure 1:**
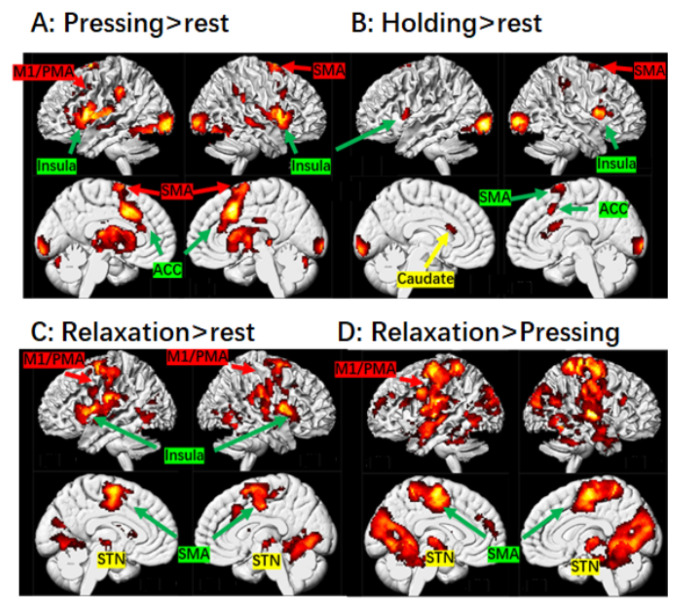
Brain regions activated during different stages of muscle control. (**A**) Brain activation during the finger pressing stage are displayed on the sagittal and axial planes. (**B**) Brain activation during the finger holding stage. (**C**) Brain activation during the relaxation stage. (**D**) Contrast map of relaxation > pressing. The voxel-wise threshold was set at *p* < 0.001 and k = 80; *p* was set at 0.005 for the holding stage. Abbreviations: ACC, anterior cingulate cortex; M1/PMA, primary motor cortex/premotor area; SMA, supplementary motor area; STN, subthalamic nucleus.

**Figure 2:**
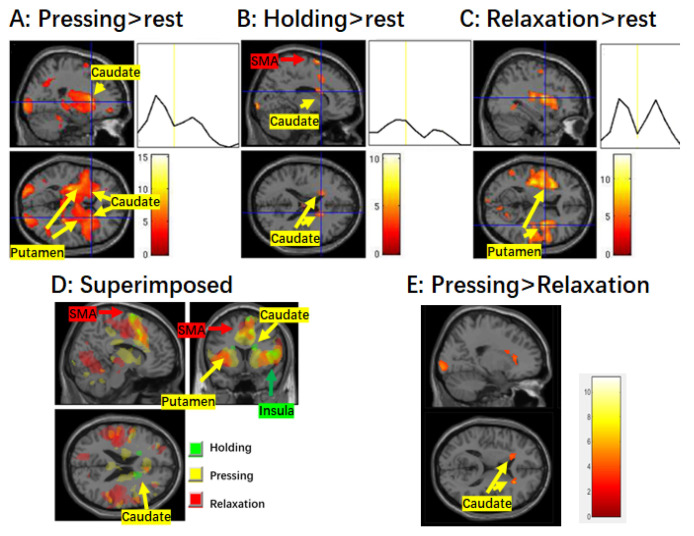
Activation in the striatum and mean time course during different stages of muscle control. (**A**) Brain activation during the finger pressing stage. Significant activation in both the caudate and putamen is shown. There is an obvious peak in the average time course of the caudate corresponding to the pressing stage. (**B**) Brain activation during the finger holding stage. Significant activation as well as increased BOLD signal are only observed in the caudate. (**C**) Brain activation during the finger relaxation stage. Significant activation is only observed in the putamen, and not the caudate. Note that there are two obvious peaks in the time course of the putamen corresponding to the pressing and relaxation of the finger, respectively. (**D**) Superimposed map of the three stages. (**E**) Contrast map of pressing > relaxation. The voxel-wise threshold was set at *p* < 0.001 and k = 80 for the finger pressing and relaxation stages, but *p* < 0.005 for other contrasts. Abbreviations: SMA, supplementary motor area.

Detailed information regarding the activation of brain regions during different stages of muscle control is presented in Table 1, and brain activation is illustrated in Figure 1 and Figure 2.

During the pressing stage, significant activation was observed in the broad motor-related cortical regions, including the left M1/PMA, SMA/ACC, bilateral insula, etc. (Figure 1). In the basal ganglia, the bilateral caudate and putamen of the striatal regions and the globus pallidus were significantly activated (Figure 2). These activation patterns were similar to those found by Sugawara et al. [5] and Spraker et al. [8].

During the steady holding stage, significant activation in the bilateral SMA/ACC, insula, and caudate (but not the putamen) were observed (Figure 1 and Figure 2).

During the relaxation stage, in the cortical regions, activation in the SMA/ACC, bilateral insula, etc., was observed. In the subcortical basal ganglia, activation in bilateral putamen and STN were observed. Activation in the STN was not evident during the pressing and holding stages. No significant activation in the caudate was detected.

## 4. Discussion

In the present study, we examined the neural correlates of different stages of muscle control while participants were instructed to press, to hold, and to relax their right index finger. Our findings revealed that there were differential cortical activation patterns among the pressing, holding, and relaxation stages. A widely distributed network was involved not only during the pressing stage, but also during the relaxation stages, only parts of which were involved in the holding stage. Furthermore, the subcortical basal ganglia played crucial roles in muscle control, with their substructures, including the caudate and putamen of the striatum, as well as the STN, exhibiting different contributions across different stages of muscle control.

### 4.1. Cortical Network

A widely distributed cortical network was engaged during the pressing and relaxation stages, only parts of which were involved in the holding stage. These findings underscore that even simple muscle control is not simple at the neural level, and intricate automatic sensory–motor integration between cortical regions such as the SMA and insula is needed to provide information on self-awareness, etc., particularly when coordinating relative muscle movements, including both the pressing and relaxation stages, whereas only minimal effort is needed to sustain muscle contraction.

#### 4.1.1. M1/PMA

Our findings revealed that the M1 and PMA were activated during both the pressing and relaxation stages, which is consistent with the findings of Toma et al. [17]. Toma et al. [17] proposed that a transient signal increase in the M1 associated with voluntary muscle relaxation suggests two possibilities in terms of the types of activated neurons: corticospinal projection neurons targeting spinal inhibitory interneurons and intrinsic inhibitory interneurons. We concur with their argument. However, we did not observe significant activation in the M1/PMA during the holding stage, which was only observed when lowering the voxel-wise threshold to *p* = 0.05. This result implies that minimal effort is required to sustain muscle contraction, aligning with Kuhtz-Buschbeck et al.’s observation that brain activation was rather weak during normal holding [9].

#### 4.1.2. SMA

Interestingly, we observed that the SMA was activated across all the three stages of muscle control. A growing body of evidence suggests that the SMA is functionally subdivided into the rostral (pre-SMA) and caudal (SMA proper) in humans [41]. While both areas are involved in motor functions, the pre-SMA is primarily associated with more complex processes such as learning, cognitive processes, and perception. The SMA proper is directly connected to the M1 and spinal cord [42], and thus, is thought to function either in parallel with or hierarchically superior to the M1. Conversely, the area pre-SMA receives strong inputs from the prefrontal cortex and projects to the somatotopic representation of the upper limb in the SMA proper, but lacks a direct connection to the M1 and spinal cord [41]. Therefore, it is supposed to play a superior role to the SMA proper [41]. In this study, as displayed in Figure 1D, significantly greater activation was observed not only in the rostral but also in the caudal part of the SMA during muscle relaxation than during muscle contraction, which suggests that both the pre-SMA and SMA proper areas may play roles in motor inhibition, in line with Toma et al. [17]. However, visual inspection revealed that only the caudal SMA was activated in the holding stage, indicating a reduced requirement for higher motor control. This could be attributed to the absence of any adjustments, resulting in minimal bottom-up updates for higher-level processing during the holding stage.

#### 4.1.3. Insula

The activation of the insula has been consistently reported in multiple studies on inhibition control [34,43]. However, contrary to previous findings, insula activation was observed throughout all three stages in the present study. These findings challenge the notion of a general role of the insula in inhibition. It is suggest that the activation in the insular cortex may reflect the body representation [44] for further movement decision-making. The insula processes a wide range of sensory signals arising from the body [45], and is related to the awareness of perception, the sense of limb ownership, self-awareness of one’s actions, and sensory–motor integration [45,46,47]. For example, patients with right posterior insular lesions may have the feeling that their contralesional limb(s) do not belong to their body or even belong to another person [48]. Tinaz et al. argued that the insula is a major hub within the limbic circuits, and the interaction between the insula and dorsomedial frontal cortex is involved in generating intentional movements. In doing so, it provides the impetus to the dorsomedial frontal cortex to initiate and sustain movement [45]. Indeed, the insula is implicated in coordinating complex articulatory movements in patients with damage to the insula [49]. Therefore, it can be inferred that that the insula is automatically involved during the pressing, holding, and relaxation stages to provide proprioception, information on the current state of the muscle, etc., and is related to sensory–motor integration for completing the tasks.

### 4.2. Subcortical Network

#### 4.2.1. Striatum

During the holding stage, we observed significant activation only in the bilateral caudate, while no significant activation was found in the putamen (Figure 2). Subsequently, a direct comparison of the BOLD signal between the pressing and relaxation stages revealed similar activation patterns in the caudate to those observed during the holding stage (Figure 2E), consistent with previous findings by Vaillancourt et al. [21] and Spraker et al. [8], indicating greater activity in the bilateral caudate nucleus during force generation compared to force relaxation. It is important to note that during both the pressing and holding stages, participants needed to keep their muscles contracted. Therefore, there is continual control of the alpha motor neurons. Taken together, these consistent results strongly suggest that the caudate nucleus plays an important role in activating the alpha motor neurons.

During the relaxation stage, only activation of the putamen, rather than the caudate, was observed, indicating that the putamen plays a crucial role in muscle relaxation or the deactivation of alpha motor neurons. Indeed, previous studies have consistently implicated the putamen in inhibition tasks such as motor NoGo tasks [50,51] and a non-motor counting NoGo task [28].

The bilateral caudate and putamen were extensively implicated in the pressing stage, a finding consistent with Spraker et al. [8]. However, it is unclear whether they play distinct roles in muscle control. To explain these results, it is imperative to consider the following factors: (1) Successful smooth movements require both intended movements and inhibition. (2) At least two groups of muscles that are attached to a joint are involved in accomplishing a movement; while one group of muscles contracts, the other antagonistic muscle group must be relaxed, or at least restricted from contraction. (3) It is crucial to avoid conflicting actions from other parts of the body. Taking cortical activation into consideration, we postulate that both the caudate and putamen are vital for smooth movement, as their coactivation provides coordinated, top-down control, ensuring that a movement is carried out smoothly as intended, not jerkily or with unintended slowness, and without interference from antagonistic muscles or conflicting action from other parts of the body. A similar explanation may apply to the relaxation stage. Pairwise comparison between the pressing and relaxation stages did not reveal significant difference between pressing and relaxation in most of the striatum, but only showed differences in small fractions of the caudate and STN. It is possible that patients with conditions such as Huntington’ disease and Parkinson’s disease exhibit unequal dysfunctions between the caudate and putamen, disrupting their elaborate cooperation, which leads to abnormal behavior. In fact, nearly half of people in the early stages of Parkinson’s disease already have signs of neurodegeneration in the caudate [52], but not in the putamen.

#### 4.2.2. STN

During the relaxation stage, participants were instructed to simply relax their muscles. Activation in the STN was observed in this process, consistent with a non-motor Count NoGo task with nearly identical coordinates [28]. This finding aligns with the basal ganglia model, where the STN is part of the indirect pathway as well as the hyperdirect pathway for inhibiting a behavior [23,24,31,32,34]. However, Spraker et al. [8] did not find significant activation in the STN. It is worth noting that in Spraker et al.’s study, the force generation task involved a precisely controlled force generation sequence repeated five times during 30 s; similarly, the precisely controlled force relaxation sequence was also repeated five times during 30 s. Clearly, both muscle contractions (indeed, for the majority of the 30 s) and relaxation were essential for these two conditions. In fact, the authors reported that the areas active during force generation were also active during force relaxation [8], potentially making it difficult to detect STN activation.

### 4.3. Limitations

Firstly, in this study, participants were instructed to perform a simple pressing task with cues, indicating that this is an externally guided task. It is expected that higher cognitive processing of visual stimuli should be engaged. However, we did not find significant involvement of the middle frontal gyri after correction, which are part of the higher cognitive network. This suggests that although this potential confounding factor cannot be entirely excluded, its impact appears to be limited. It is plausible that different activation patterns may emerge with internally guided tasks. Secondly, the sample size was 10. Typically, movement-related BOLD signals exhibit a large effect size compared to other cognitive tasks (especially compared to rest); therefore, relatively few participants are required for reliable results. For instance, previous studies have utilized 5 [53], 6 [3], 8 [9], 9 [54], 11 [21], and 12 [8] participants, respectively. Consistent with these studies, and as shown in Table 1, significant activation was also observed in movement-related cortical regions with a conservative, FWE-corrected threshold in the present study. Nevertheless, further studies with larger sample sizes are warranted.

## 5. Conclusions

To the best of our knowledge, this is the first fMRI study to investigate the neural correlates of different stages of muscle control. Our findings indicate that (1) different cortical activation patterns were observed during the pressing, holding, and relaxation stages of muscle control; (2) the subcortical basal ganglia played crucial roles in muscle control; and their substructures, including the caudate, putamen, and STN, played distinct roles in different stages of muscle control. We hence provide one piece of direct evidence that the caudate and putamen may play different but coordinated roles in muscle control, and support the direct/indirect pathway models of the basal ganglia in the regulation of movement. Our findings highlight that even seemingly simple muscle control involves complex automatic sensory–motor integration processes, particularly when coordinating relative movements, including both muscle contraction and relaxation. Further studies are warranted to determine whether the same neural mechanism can be applied to spontaneous/internally guided muscle control.

## Figures and Tables

**Table 1:** Brain regions activated during different stages of muscle control.

Conditions	Location	Side	MNI Coordinates			Cluster Size	T Score
			x	y	Z		
Pressing > rest							
	M1/PMA	L	−34	−34	36	790	6.42 **
		R	58	−34	24	1417	8.04 ***
	SMA	L/R	0	16	44	2026	12.28 ***
	ACC	L/R	8	24	28	1813	6.69 *
		R	6	−38	50	215	4.79
	Insula/IFG	L	−32	2	14	1187	15.22 ***
		R	36	8	12	1259	8.06 ***
	Caudate	L	−16	6	20	326	6.6 ^†^
		R	22	18	6	595	8.86 *
	Putamen	L	−28	−20	4	995	9.43 **
		R	24	16	8	1000	8.11 **
	Pallidum	L	−26	−6	−2	248	7.67
		R	24	−2	−6	277	5.53
	MOG/IOG	L	−22	−90	−6	1386	9.17 ***
		R	38	−72	−12	842	7.09 **
	FG	L	−32	−72	−18	566	6.59 *
		R	44	−62	−22	530	7.46 *
	Thalamus	L/R	−22	−18	0	1335	7.16 ***
	MFG	L	−30	46	28	144	5.56
		R	38	44	30	343	4.85
	Cerebellum	L	−32	−74	−20	589	6.55 *
		R	38	−66	−24	1723	9.20 ***
Holding > rest							
	SMA	R	14	10	72	182	5.81
	ACC	R	12	12	48	139	4.65
	Insula/IFG	R	50	8	10	647	10.19 **
		L	−40	6	14	181	4.3 ^†^
	PoCG	R	64	−30	46	166	5.11
	Caudate	R	24	24	10	321	5.51 ^†^
		L	−18	16	22	91	4.68 ^†^
	MOG	L	−24	−90	−4	1128	10.43 ***
		R	26	−92	0	924	8.59 **
Relaxation > rest							
	M1/PMA	L	−48	−22	26	2082	8.86 ***
		R	58	14	32	938	6.68 **
	SMA	L/R	−10	−8	52	2835	11.26 ***
	ACC	L/R	12	−8	50	1411	13.13 **
	Insula/IFG	L	−40	14	8	1702	10.22 ***
		R	34	−2	12	1845	9.06 ***
	Putamen	L	32	2	10	563	8.58 ^‡^
		R	−30	8	−6	543	8.64 ^‡^
	STN	L/R	4	−14	−4	194	7.39 **
	LG/PhG/FG	R	20	−70	−2	1803	7.27 ***
		L	−22	−70	−4	1138	8.87 **
	PCG	L	−20	−10	66	949	6.01 **
		R	26	−18	16	1262	7.55 **
	MTG	L	−52	−62	4	656	5.61 *
	Cuneus	L	−14	−76	20	219	5.25
	MFG	L	−24	38	26	354	4.58
		R	34	38	24	132	4.41
	Cerebellum	L	−34	−38	−30	132	4.49
		R	14	−52	−18	1114	5.98 **
	STG	L	−46	32	22	1283	7.46 **
		L	52	−24	16	1186	6.88 **

Abbreviations. L, left, R, right; ACC, anterior cingulate cortex; FG, fusiform gyrus; IFG, inferior frontal gyrus; IOG, inferior occipital gyrus; LG, lingual gyrus; M1/PMA, primary motor cortex/premotor area; MFG, middle frontal gyrus; MOG, middle occipital gyrus; MTG, middle temporal gyrus; PCG, pre-central gyrus; PhG, parahippocampal gyrus; PoCG, post-central gyrus; SMA, supplementary motor area; STG, superior temporal gyrus; STN, subthalamic nucleus. * *p* < 0.05, ** *p* < 0.01, *** *p* < 0.001, FWE corrected at cluster-level; ^†^ *p* < 0.05, ^‡^ *p* < 0.01, small volume correction.

## Data Availability

All data is available at https://osf.io/xjg2z/ (accessed on 18 April 2024).
